# WORK ENVIRONMENTS AMONG OLDER WORKING CAREGIVERS

**DOI:** 10.1093/geroni/igz038.798

**Published:** 2019-11-08

**Authors:** Dawn C Carr, Miles G Taylor, Kendra Jason, Chivon A Mingo, Tiffany R Washington

**Affiliations:** 1 Florida State University, Tallahassee, Florida, United States; 2 University of North Carolina at Charlotte, Charlotte, North Carolina, United States; 3 Georgia State University, Atlanta, Georgia, United States; 4 University of Georgia, Athens, Georgia, United States

## Abstract

Many older workers balance paid work with care work. Working caregivers face unique challenges that make them more likely to leave the work force. However work environments may be more or less accommodating to their needs, and in addition, they may need to work for financial reasons. Current research on working caregivers has not explored: a) the work environments of older working caregivers; b) whether particular work environments are likely to influence whether caregivers stop working; and c) whether these effects vary by type of care work (spousal versus parental). This study addresses these gaps. Using data drawn from the 2008-2014 waves of the Health and Retirement Study, we used latent class analysis to develop a typology of work environments of individuals 51-75 who are engaged in paid work. Four classes of work environments emerged: A) balanced, supportive work environments (34%); B) average environments with high job lock (30%); C) poor, unsupportive work environments (21%); and D) highly accommodating, stressful jobs (14%). Logistic regression results showed those in group D were less likely than all other groups to leave their jobs. In addition, relative to spousal caregivers, parental caregivers in class A were significantly more likely to leave the labor force. Results suggest that caregivers may be more likely to continue engaging in paid work in supportive work environments, and work environments may be more likely to retain older working caregivers by identifying ways to help them meet their work needs and maintain their caregiving roles.

